# Insecticide resistance levels and associated mechanisms in three *Aedes aegypti* populations from Venezuela

**DOI:** 10.1590/0074-02760220210

**Published:** 2023-06-23

**Authors:** Yasmin Rubio-Palis, Nicole Dzuris, Christopher Sandi, Rita Lucrecia Vizcaino-Cabarrus, Claudia Corredor-Medina, Jesús Alberto González, Audrey E Lenhart

**Affiliations:** 1 Universidad de Carabobo, Instituto de Investigaciones Biomédicas, Maracay, Venezuela; 2 US Centers for Disease Control and Prevention, Center for Global Health, Division of Parasitic Diseases and Malaria, Entomology Branch, Atlanta, GA, USA; 3 Ministerio del Poder Popular para la Salud, Dirección General de Salud Ambiental, Dirección de Control de Vectores, Maracay, Venezuela

**Keywords:** Aedes aegypti, metabolic detoxification, *kdr* mutations, V410L, F1534C, V1016I

## Abstract

**BACKGROUND:**

The massive use of insecticides in public health has exerted selective pressure resulting in the development of resistance in *Aedes aegypti* to different insecticides in Venezuela. Between 2010 and 2020, the only insecticides available for vector control were the organophosphates (Ops) fenitrothion and temephos which were focally applied.

**OBJECTIVES:**

To determine the state of insecticide resistance and to identify the possible biochemical and molecular mechanisms involved in three populations of *Ae. aegypti* from Venezuela.

**METHODS:**

CDC bottle bioassays were conducted on *Ae. aegypti* collected between October 2019 and February 2020 in two hyperendemic localities for dengue in Aragua State and in a malaria endemic area in Bolívar State. Insecticide resistance mechanisms were studied using biochemical assays and polymerase chain reaction (PCR) to detect *kdr* mutations.

**FINDINGS:**

Bioassays showed contrasting results among populations; Las Brisas was resistant to malathion, permethrin and deltamethrin, Urbanización 19 de Abril was resistant to permethrin and Nacupay to malathion. All populations showed significantly higher activity of mixed function oxidases and glutathione-S-transferases (GSTs) in comparison with the susceptible strain. The *kdr* mutations V410L, F1534C, and V1016I were detected in all populations, with F1534C at higher frequencies.

**MAIN CONCLUSION:**

Insecticide resistance persists in three *Ae. aegypti* populations from Venezuela even in the relative absence of insecticide application.

*Aedes aegypti* is the principal vector of several viruses including dengue, chikungunya, Zika and yellow fever.^1^ This species originated in Africa and was originally a tree-hole forest mosquito,^2^ spread to other continents through trade, and subsequently adapted to urban environments where the females oviposit in artificial containers.^3^ More recently, *Ae. aegypti* has colonized rural areas throughout the world^4^ ^,^ ^5^ ^,^ ^6^ and even remote isolated forest areas in southern Venezuela,^7^ broadening the risk of arbovirus transmission.

According to World Health Organization (WHO),^1^ dengue incidence has increased considerably in recent decades; most cases are asymptomatic or mild, or misdiagnosed as a febrile illness, and hence, the actual number of cases are under-reported. It has been estimated that 3.9 billion people are at risk of infection in 129 countries^8^ with an estimated global burden of 390 million dengue infections per year.^9^ At present, prevention and control of the transmission of dengue viruses is based heavily on vector control. Many efforts have been directed at the reduction of larval habitats and environmental sanitation with community participation, although these approaches can be challenging to sustain, especially in areas with socio-political-economic crises such as in Venezuela.

The use of chemical insecticides has been the main intervention for the control of *Ae. aegypti* in Venezuela for over six decades. Dichlorodiphenyltrichloroethane (DDT) was widely used from the 1950’s until the mid-1970’s for the control of *Ae. aegypti* (yellow fever) and anophelines (malaria). With the availability of the yellow fever vaccine and an efficient vaccination program, the use of DDT was discontinued in urban areas, but its use continued in rural areas for the control of vectors of malaria parasites until the mid-1990’s and was finally banned in 2001.^10^

In 1989-1990, the first major outbreak of dengue and dengue hemorrhagic fever (DHF) was reported in Venezuela, with the first cases reported in Maracay, Aragua State and from there expanding to many urban areas in the country.^11^ ^,^ ^12^ Subsequently, continuous outbreaks of dengue have been reported from Venezuela each year, with the simultaneous circulation of DENV1, DENV2, DENV3 and DENV4.^13^ Although reduction of larval sources such as water tanks, cisterns, and used tires can help to suppress vector populations, its implementation has been sporadic while the use of insecticides has been extensive and intense. The primary insecticides used in Venezuela are organophosphates (OPs), such as temephos for larval control, and malathion in thermal foggers and ultra-low volume sprays for adult mosquito control. Additionally, pyrethroids were often incorporated into vector control programs. Since 2010, only fenitrothion (an OP) and temephos were available in limited quantities and applied in a focal manner: in and around houses with confirmed severe dengue cases.

The massive use of insecticides in public health has exerted selective pressure resulting in the development of resistance in *Ae. aegypti* to different insecticides in Venezuela as well as all over the world.^14^ Resistance to DDT in *Ae. aegypti* was reported for the first time by Quarterman and Schoof^15^ in Caracas, followed by a study conducted by Mouchet^16^ in seven populations along the northern part of the country where resistance to DDT, dieldrin, and hexachlorocyclohexane (HCH) was reported. Mazzarri and Georghiou^17^ detected resistance to OPs, carbamates, and pyrethroids in the states of Aragua and Falcón, even though control programs using these insecticides had only recently been implemented. Álvarez et al.^18^ ^,^ ^19^ reported resistance to malathion, permethrin, and deltamethrin in populations of *Ae. aegypti* from western Venezuela, whereas Molina de Fernández et al.^20^ showed for populations from different parts of the country that resistance to malathion was focal. More recently, Bastidas et al.^21^ reported resistance to DDT in seven populations of *Ae. aegypti* from Aragua State, but all were susceptible to deltamethrin.

Resistance to insecticides is often due to an increase in metabolic activity and/or alteration of the insecticide target site. Three major groups of enzymes are often involved in metabolic resistance: carboxylesterases, cytochrome p450 monooxygenases, and glutathione-S-transferases (GSTs). In general,^22^ esterases are associated with resistance to OPs, carbamates, and pyrethroids; the monooxygenases are involved in the metabolism of pyrethroids and detoxification of OPs and to lesser degree methyl-carbamates; and GSTs metabolize DDT to less toxic products and play a secondary role in resistance to pyrethroids and OPs.

Biochemical and synergistic assays conducted on *Ae. aegypti* in different parts of Venezuela reported high levels of esterase activity associated with resistance to temephos, malathion, chlorpyrifos, and pirimiphos methyl.^17^ ^,^ ^18^ ^,^ ^23^ ^,^ ^24^ Other enzymes detected in populations of *Ae. aegypti* are overexpression of oxidases and GSTs associated with resistance to DDT.^21^

Target site mechanisms involved in resistance to DDT and pyrethroids are mutations in the voltage-gated sodium channel (VGSC) of the nerve cell membrane. These mutations cause a reduction in the sodium channel sensitivity to the insecticides resulting in knockdown resistance (*kdr*) where the mosquito does not lose coordination immediately after exposure to the insecticide. Saavedra-Rodríguez et al.^25^ reported very low frequencies of the mutations V1016I, I1011V, and I1011M among nine populations from Venezuela, whereas Álvarez et al.^26^ found higher frequencies for the mutation F1534C than for the mutation V1016I in natural populations of *Ae. aegypti* from five states. Monitoring of insecticide resistance and the mechanisms involved are a priority within the vector control program due to hyperendemicity of dengue in urban areas and the risk of outbreaks extending to rural and remote areas. The objectives of the present study were to determine the state of insecticide resistance and to identify the possible biochemical and molecular mechanisms involved in three populations of *Ae. aegypti* from Venezuela: two sites hyperendemic for dengue, and the third located in a gold mining area which presented the risk of both *Aedes*-borne arboviruses as well as malaria.

## MATERIALS AND METHODS

**Fig. 1:**
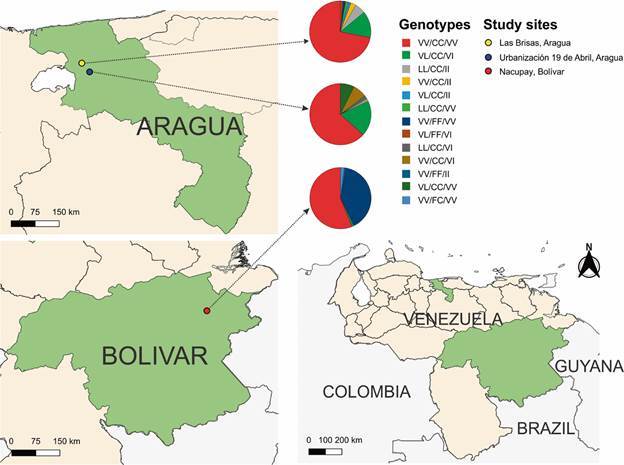
relative location of study sites and genotype frequency for the *kdr* mutations V410L, F1534C, and V1016I in three populations of *Aedes aegypti* from Venezuela. Natural Earth raster (public domain: https://www.naturalearthdata.com).

*Study sites* - *Aedes aegypti* immature stages were collected between October 2019 and February 2020 in three localities (Fig. 1). Two were neighborhoods hyperendemic for dengue in the Maracay Metropolitan Area (estimated population: 1,212,981),^27^ Aragua State and located 12.2 km apart: Las Brisas, Girardot Municipality (10º 17’ 3” N; 67º 24’ 21” W, altitude 470 m), with approximately 400 houses and a population of 1,600, and Urbanización 19 de Abril, Santiago Mariño Municipality (10º 13’ 24” N; 67º 30’ 56” W, altitude 436 m), with approximately 462 houses and a population of 1,848. The area is characterized by a mean annual temperature of 25.5ºC, 75% relative humidity and 910 mm of rainfall.^28^ The third locality, Nacupay, El Callao Municipality, Bolívar State (7º 20’ 36” N; 61º 49’ 55” W, 167 m), is a gold mining neighborhood established around 1998 south of the town of El Callao, at the margins of the Yuruari River; this region is endemic for malaria with relative recent colonization by *Ae. aegypti* (< 20 years). Nacupay has about 180 houses and an estimated population of 720 and is characterized by an annual mean temperature of 26.2ºC, 81.9% relative humidity and annual rainfall of 1,276 mm.^29^ The distance between Las Brisas and Nacupay is 950 km. The type of houses in the three locations are similar: brick, plastered and painted walls; galvanized sheet or concrete roofs; cement, tiled or granite floors. The relative location of study sites was plotted on a map based on images from Natural Earth raster (public domain: https://www.naturalearthdata.com). Between 2,000 and 3,000 larvae and pupae were collected from 200 L plastic containers used for water storage for domestic use in each locality and transported to the insectary of the Vector Control unit of the Directorate of Environmental Sanitation, Maracay for mosquito rearing and obtaining the next generations. Dry eggs (F1 from Aragua State and F6 from Bolívar State) were transported to the insectary at the US Centers for Disease Control and Prevention (CDC) in Atlanta, Georgia, USA and mosquitoes were reared at 27ºC and 80% relative humidity (RH). Females aged three to five days old and fed on a 10% sugar solution were used for bioassays.

*Insecticide susceptibility tests* - Bioassays were performed using the protocols described by CDC^30^ at 24.8 - 25.8ºC and 17.5 - 20% RH. For each insecticide tested (Chem Service Inc, West Chester, PA), four replicates and one control were analyzed. Table I shows the insecticides tested for each *Ae. aegypti* strain, number of mosquitoes exposed, generation (F), diagnostic doses and percent mortality at 30 minutes exposure (diagnostic time). Malathion and deltamethrin bioassays for Las Brisas population were repeated on two consecutive days, and as results were similar, the data were pooled. WHO^31^ criteria were used to determine the status for each population and each insecticide tested: R = resistant (mortality < 90%); C = resistance to be confirmed (mortality = 90-97%); S = susceptible (mortality ≥ 98%). Alive and dead mosquitoes were kept at -20ºC for molecular studies, and non-insecticide-exposed mosquitoes were stored at -20ºC for biochemical assays. Bioassays were not conducted using a susceptible reference strain.

**TABLE I:** Insecticides tested, diagnostic doses, number of mosquitoes exposed and percent mortality at the diagnostic time of 30 minutes for three *Aedes aegypti* populations from Venezuela

Locality	Insecticide tested	Diagnostic dose (µg/mL)	#Mosq exposed	generation (F)	Percent mortality	Status^*a*^
Las Brisas	Malathion	50	214	F1	78.5	R
	Malathion + PBO^*b*^	50	100	F2	93.0	C
	Permethrin	15	122	F1	2.5	R
	Permethrin 2X	30	113	F1	24.8	R
	Permethrin + PBO^*b*^	15	100	F2	100	S
	Deltamethrin	10	201	F1	52.7	R
	Deltamethrin 2X	20	105	F1	100	S
	Lambdacyhalothrin	10	100	F2	100	S
Urbanización 19 de Abril	Malathion	50	112	F2	98.2	S
	Permethrin	15	104	F2	74.0	R
	Cypermethrin	10	102	F2	100	S
Nacupay	Malathion	50	121	F6	89.3	R
	Permethrin	15	100	F7	100	S
	Deltamethrin	10	100	F7	100	S
	Alphacypermethrin	10	100	F7	100	S
	Lambdacyhalothrin	10	100	F7	100	S

*a*: R = resistance (mortality < 90%); C = resistance to be confirmed (mortality = 90-97%); S = susceptible (mortality ≥ 98%)^31^; *b*: PBO = Piperonyl butoxide.

*Synergist bioassays* - The action of the synergist piperonyl butoxide (PBO), which inhibits oxidase activity, was investigated by exposing adult females of Las Brisas population 1 h prior to exposure to malathion or permethrin (Table I) following the CDC protocol.^30^ Due to limited numbers of mosquitoes, we were unable to test for the inhibitory activity of additional synergists.

*Biochemical assays* - To measure levels of detoxification enzymes, proteins, and the acetylcholinesterase insensitivity, assays were conducted according to published protocols.^32^ ^-^ ^37^ All assays were conducted on 60 female mosquitoes each from Las Brisas, Urbanización 19 de Abril and Nacupay. Additionally, 30 female Rockefeller (susceptible strain) mosquitoes were run for comparison. Each individual whole mosquito was homogenized in 100 µL of KPO_4_ buffer (pH 7.2; pH adjusted with phosphoric acid) using a pestle and battery-operated homogenizer. The homogenate was then diluted to 2 mL. For all assays, each mosquito was run in triplicate. Plates were read using a SpectraMax M5e plate reader and SoftMax Pro 7 software (Molecular Devices, Sunnyvale, CA). Unless otherwise stated, all chemicals were purchased from Sigma-Aldrich (St. Louis, MO).

The acetylcholinesterase and insensitive acetylcholinesterase assays were run simultaneously on separate plates and the difference in color was compared between the two assays. For these assays, three solutions were made; ATCH (75 mg acetylthiocholine iodine dissolved in 10 mL acetone, and 90 mL KPO_4_ added), ATCH with propoxur (75 mg acetylthiocholine iodide, and 21 mg propoxur dissolved in 10 mL acetone, and 90 mL KPO_4_ buffer added), and DTNB [13 mg 5,5’-Dithiobis-(2-nitrobenzoic acid) dissolved in 100 mL KPO_4_ buffer]. For the insensitive acetylcholinesterase plate, each well contained 100 µL mosquito homogenate, and 100 µL ATCH with propoxur solution, and 100 µL DTNB solution. For the acetylcholinesterase plate, each well contained 100 µL mosquito homogenate, and 100 µL ATCH solution, and 100 µL DTNB solution. After 60 minutes incubation, absorbance values for each plate were read using a 414 nm filter.

The mixed-function oxidase (MFO) assay does not measure enzymatic activity, but rather quantifies the heme group mainly associated with mixed function oxidases, including cytochrome p450s. A solution of 3,3’,5,5’-tetra-methybenzidine (TMBZ) was prepared by dissolving 50 mg of TMBZ in 25 mL methanol and adding 75 mL of 0.25 M sodium acetate buffer (pH 5.0; pH adjusted with glacial acetic acid). Each well contained 100 µL of mosquito homogenate, 200 µL TMBZ solution, followed by 25 µL of 3% hydrogen peroxide. The positive control consisted of 10 µL of cytochrome C (0.01 mg/mL) and 90 µL of KPO_4_ buffer. After a 10-minute incubation, absorbance values were read using a 650 nm filter.

For GSTs, solutions of reduced glutathione (61 mg L-glutathione reduced and 100 mL KPO_4_ buffer) and cDNB (20 mg 1-chloro-2,4’-dinitrobenzene dissolved in 10 mL acetone, and 80 mL KPO_4_ buffer added) were made. To each well, 100 µL of mosquito homogenate (after centrifugation), 100 µL reduced glutathione solution, and 100 µL cDNB were added. The plate was read immediately (T0) and after 10 minutes incubation using a 340 nm filter.

For the protein assay, a solution of protein assay dye reagent concentrate (Bio-Rad) and dH_2_O (1:4) was made. Each well contained 20 µL mosquito homogenate (after centrifugation), 80 µL KPO_4_ buffer, and 200 µL protein dye solution. After 10 minutes incubation, absorbance values for each plate were read using a 595 nm filter. Based on the Bradford method of protein quantification,^38^ a standard curve using bovine serum albumin was generated to quantify the total protein present in each mosquito. This total serves as an estimate for the size of each mosquito and corrects values obtained for all assays, apart from the acetylcholinesterase assay.

Kruskal-Wallis non-parametric tests were used to determine statistically significant differences in assay values by population. When differences were detected, Dunn’s Test was used for pairwise comparisons. Statistical analyses and figures were carried out using RStudio.^39^ ^,^ ^40^ Results were considered statistically significant at p < 0.05.

kdr allele detection

*DNA extraction* - The Quanta Biosciences Extracta^TM^ Kit was used for DNA extraction from mosquitos of Las Brisas and Urbanización 19 de Abril (phenotype permethrin resistant), Nacupay (phenotype permethrin susceptible). Each mosquito was placed in a sterile 0.2 mL tube and 25 μL extraction buffer was added, followed by an incubation at 95ºC for 30 min in a C1000 Bio-Rad CFX 96 Touch^TM^ Real-Time System thermocycler. At the end of the incubation, 25 μL of stabilization buffer was added. The concentration and quality of each DNA sample were determined in a NanoDrop^TM^ 2000/2000c spectrophotometer (Thermo Fisher Scientific).

*Detection of mutations V410L, F1534C and V1016I and genotyping* - Allele-specific polymerase chain reaction (PCR) was carried out in a C1000 Bio-Rad CFX 96 Touch^TM^ Real-Time System thermocycler using the primers for V410L, V1016I and F1534C mutations (Table II) to determine the genotypes for each mutation by melting curve analysis following modified protocols from those described by Saavedra-Rodríguez et al.^25^ ^,^ ^41^ and Yanola et al.^42^

**TABLE II:** Primer sequencies used in *kdr* genotyping.

Mutation	Primer name	Sequence (5’ - 3’)
V410L	Val410fw	GCG GGC AGG GCG GCG GGG GCG GGG CCA TCT TCT TGG GTT CGT TCT ACC GTG
	Leu410fw	GCG GGC ATC TTC TTG GGT TCG TTC TAC CAT T
	410rev	TTC TTC CTC GGC GGC CTC TT
F1534C	Cys1534fw	GCG GGC AGG GCG GCG GGG GCG GGG CCT CTA CTT TGT GTT CTT CAT CAT GTG
	Phe1534fw	GCG GGC TCT ACT TTG TGT TCT TCA TCA TAT T
	1534rev	TCT GCT CGT TGA AGT TGT CGA T
V1016I	Val1016fw	GCG GGC AGG GCG GCG GGG GCG GGG CCA CAA ATT GTT TCC CAC CCG CAC CGG
	Ile1016fw	GCG GGC ACA AAT TGT TTC CCA CCC GCA CTG A
	1016rev	TGA TGA ACC SGA ATT GGA CAA AAG C

The methodology described by Saavedra-Rodríguez et al.^41^ for the V410L mutation was modified as follows; the reaction mixture (20 μL) contained 9.9 μL of Quantabio PerfeCta SYBR Green Supermix (95054), 0.1 μL of each fw primer and 0.2 μL rev primer and 1.0 μL of DNA. The amplification consisted of 95ºC for 3 min (activation step), followed by 40 cycles at 95ºC for 10 s, 60ºC for 10 s and 72ºC for 30 s, and a final extension step of 10 s at 95ºC. The melting curves were determined from 65ºC to 95ºC, with an increase of 0.2ºC every 10 s, where a simple peak at 80ºC corresponded to the homozygous mutant type (Leu410/Leu410), two peaks at 80ºC and 83ºC corresponded to the heterozygote (Leu410/Val410) and a single peak at 83ºC corresponded to the homozygous wild type (Val410/Val410).

For the F1534C mutation, the modified methodology described by Yanola et al.^42^ was followed; the reaction mixture (18 μL) contained 9 μL of Quantabio PerfeCta SYBR Green Supermix (95054), 0.65 μL of the primer Cys1534fw, 0.6 μL of each of Phe1534fw and F1534rev primers and 2 μL of the DNA. The amplification consisted of 95ºC for 3 min, followed by 37 cycles at 95ºC for 10 s, 57ºC for 30 s and 72ºC for 30 s, and 95ºC for 10 s. The melting curves were determined from 65ºC to 95ºC, with an increase of 0.5ºC every 5 s, where a simple peak at 82ºC corresponded to the homozygous mutant type (Cys1534/Cys1534), two peaks at 78ºC and 82ºC corresponded to the heterozygote (Phe1534/Cys1534) and a single peak at 78ºC corresponded to the homozygous wild type (Phe1534/Phe1534).

The Saavedra-Rodríguez et al.^25^ modified methodology was followed for the V1016I mutation; the reaction mixture (18 μL) contained 8 μL of Quantabio PerfeCta SYBR Green Supermix (95054), 0.34 μL of primer Val1016fw and 0.4 μL of each of primers Ile1016fw and 1016rev plus 2 μL of DNA. The amplification consisted of 95ºC for 3 min, followed by 35 cycles at 95ºC for 10 s, 60ºC for 10 s and 72ºC for 30 s, and a final extension step of 10 s at 95ºC. The melting curves were determined from 65ºC to 95ºC, with an increase of 0.2ºC every 10 s, where a simple peak at 76ºC corresponded to the homozygous mutant type (Ile1016/Ile1016), two peaks at 76ºC and 83ºC corresponded to the heterozygote (Val1016/Ile1016) and a single peak at 83ºC corresponded to the homozygous wild type (Val1016/Val1016). Positive and negative (ddH_2_O) controls were run for all PCR assays.

*Allele frequencies and linkage disequilibrium analysis* - The allele frequency of each mutation (*p*) was calculated as the sum of two times the number of mutant homozygotes and the number of heterozygotes, divided by 2*n*, where *n* is the number of mosquitoes analyzed. The 95% confidence interval (CI) was calculated using the following formula:

$$\frac{2np+z^{2}\pm \;z\sqrt{{(z}^{2}+4npq)}}{2\;(n+z^{2})}$$

The HardyWeinberg package^43^ ^,^ ^44^ was used to calculate the Hardy-Weinberg equilibrium (HWE). The HWE was expressed as Wright’s inbreeding coefficient F_IS_ ^45^ and a χ^2^ test was used to test the null hypothesis F_IS_ = 0 [degrees of freedom (df) = 1].

## RESULTS

*Bioassays* - Bioassays showed that the population from Las Brisas was resistant to malathion, permethrin and deltamethrin (Table I), although mortality increased following pre-exposure to the synergist PBO before exposing to permethrin or doubling the concentration for deltamethrin; PBO pre-exposure still showed possible resistance for malathion but suggested that enzymes other than oxidases (*e.g.*, esterases), play a key role in resistance. The population of Urbanización 19 de Abril (F2) was resistant only to permethrin while Nacupay (F6) showed resistance to malathion.

**Fig. 2:**
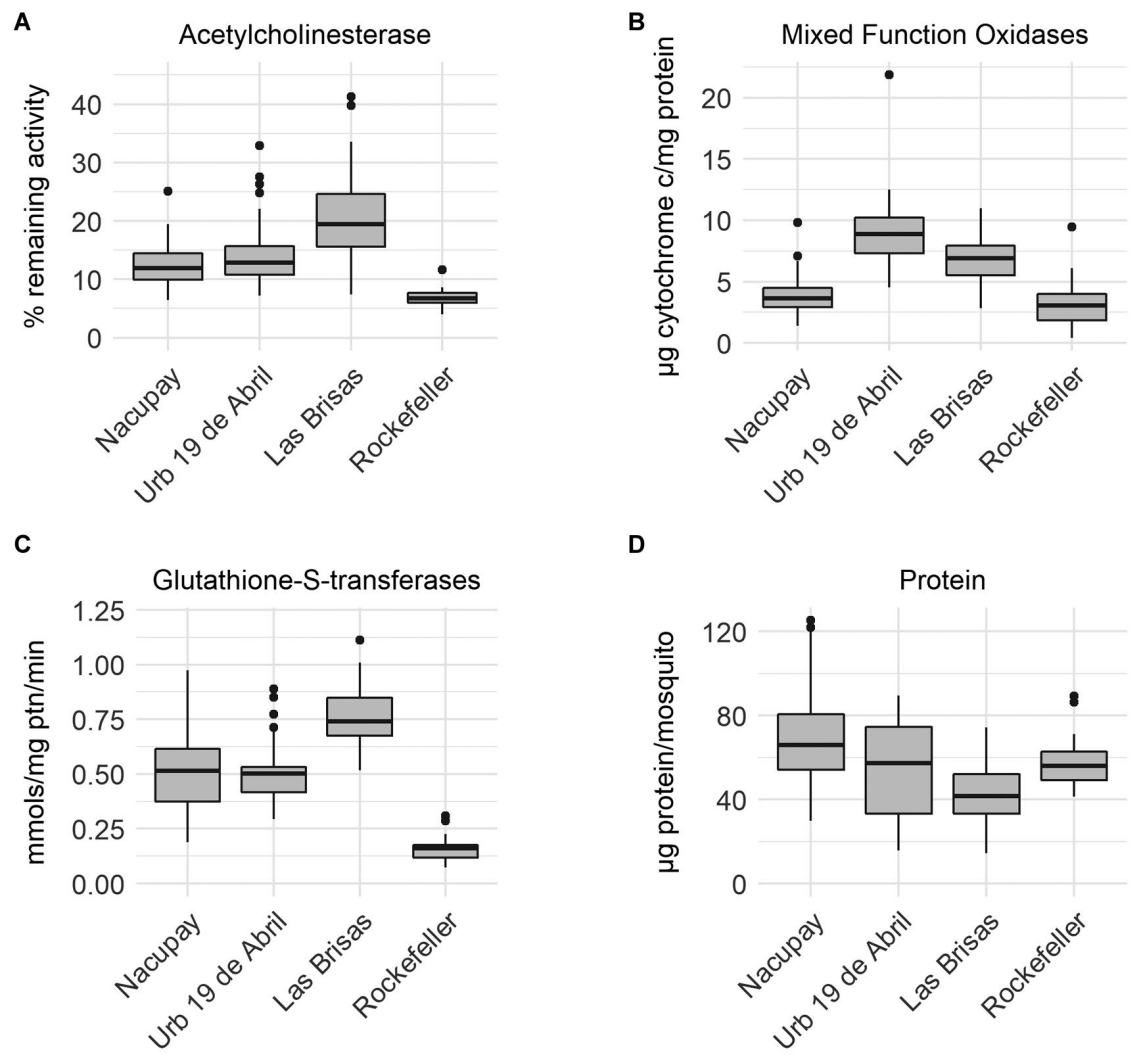
boxplots of biochemical assay results for *Aedes aegypti* populations. (A) Acetylcholinesterase. (B) Mixed Function Oxidases. (C) Glutathione-S-transferases. (D) Protein.

*Biochemical assays* - Ten percent (6 of 60) of Las Brisas samples, and 1.7% (1 of 59) of Urbanización 19 de Abril samples were higher than the 30% cut-off point for acetylcholinesterase activity remaining in the presence of propoxur (Fig. 2A).

Oxidase levels between Rockefeller (median 3.04 µg cytochrome c/mg protein) and Nacupay (median 3.64 µg cytochrome c/mg protein) were not significantly different. However, oxidase levels in Urbanización 19 Abril (median 8.90 µg cytochrome c/mg protein) and Las Brisas (median 6.92 µg cytochrome c/mg protein) were significantly higher than Rockefeller (p < 0.001). Pairwise comparisons showed significant differences between Nacupay and the two other Venezuelan populations (p < 0.001) (Fig. 2B).

Glutathione-S-transferase levels were significantly different (p < 0.001) between Rockefeller (median 0.16 mmols/mg protein/min) and all three Venezuelan populations (Urbanización 19 Abril - median 0.50 mmols/mg protein/min; Las Brisas - median 0.74 mmols/mg protein/min; Nacupay - median 0.52 mmols/mg protein/min). All pairwise comparisons between the Venezuelan populations were also statistically significant (p < 0.001) except Nacupay and Urbanización 19 Abril (Fig. 2C).

Protein levels were significantly higher (p = 0.016) in Rockefeller (median 56 µg/mosquito) than Las Brisas (41.5 µg/mosquito). However, protein levels between Rockefeller and Urbanización 19 Abril (median 57.2 µg/mosquito) and Nacupay (66.0 µg/mosquito) were not significantly different. All pairwise comparisons between the Venezuelan populations were significantly different (p < 0.05).

Although esterase assays were conducted, the results were inconsistent likely due to faulty chemicals and therefore are not reported. Due to constraints on obtaining more mosquitos it was impossible to repeat the assays.

*Kdr allele detection* - Table III shows the populations, sample sizes and numbers of mosquitoes of each genotype, the frequency of mutant alleles, the 95% confidence interval around that frequency, the inbreeding coefficient (F_IS_) and the significance (p) of the *χ*2 test.

**TABLE III:** Genotype, mutant allele frequency, 95% confidence interval (CI), inbreeding coefficient (FIS), chi-square significance (p) for mutations V410L, F1534C, and V1016I in three populations of *Aedes aegypti* from Venezuela

Mutation	Population	N	Genotype			*kdr* Frequency (95% CI)	Hardy Weinberg Disequilibrium	
			Mutant allele	Heterozygote	Wild type		FIS	p-value
V410L			LL	VL	VV			
	Las Brisas^*a*^	72	5	12	55	0.15 (0.10-0.22)	0.36	0.0025
	Urbanización 19 de Abril^*a*^	154	3	40	111	0.15 (0.11-0.19)	-0.02	0.7826
	Nacupay^*b*^	96	0	1	95	0.01 (0.00-0.03)	-0.01	0.9591
F1534C			CC	FC	FF			
	Las Brisas^*a*^	72	70	0	2	0.97 (0.93-0.99)	1	< 0.0001
	Urbanización 19 de Abril^*a*^	155	155	0	0	1.00 (0.99-1.00)	NA	NA
	Nacupay^*b*^	96	55	2	39	0.58 (0.51-0.65)	0.96	< 0.0001
V1016I			II	VI	VV			
	Las Brisas^*a*^	72	8	11	53	0.19 (0.13-0.26)	0.50	< 0.0001
	Urbanización 19 de Abril^*a*^	151	3	41	107	0.16 (0.12-0.20)	-0.03	0.684
	Nacupay^*b*^	96	1	2	93	0.02 (0.01-0.05)	0.49	< 0.0001

*a*: Las Brisas and Urbanización 19 de Abril phenotype = permethrin resistant; *b*: Nacupay phenotype = permethrin susceptible.

In general, the frequency for the mutant alleles for F1534C (CC) varied between 1 (Urbanización 19 de Abril) and 0.58 (Nacupay) while very low frequencies were found for V410L (LL) (0.01-0.15) and V1016I (II) (0.02-0.19) in all populations. Fig. 1 shows the genotype frequency in the three study sites. Double (CC/II and LL/CC) and triple mutations (LL/CC/II) were present only in the populations from Maracay with frequencies less than 3% and 6% respectively. Forty percent of mosquitoes from Nacupay were wild type for the three *kdr* alleles (VV/FF/VV). For V410L, there was deficiency of homozygotes (LL) (F_IS_ < 0) for the populations from Nacupay and Urbanización 19 de Abril while for Las Brisas there was excess of homozygotes (F_IS_ > 0). For F1534C, an excess of homozygotes (CC) was detected in all populations analyzed with fixation detected in Urbanización 19 de Abril. Although at very low frequency, the mutation V1016I had excess of homozygotes (II) in Las Brisas and Nacupay and deficiency of homozygotes in Urbanización 19 de Abril. There was HWE only for the mutant alleles LL of the populations Nacupay and Urbanización 19 de Abril and II of Urbanización 19 de Abril.

## DISCUSSION

The Venezuelan vector control program has relied mainly on the use of insecticides, with the introduction of DDT in 1946 for the control of *Anopheles* mosquitoes,^46^ and later for the control of *Ae. aegypti* in urban areas, followed by the introduction of the OPs temephos and malathion in 1970 against larvae and adults of *Ae. aegypti*,^17^ ^,^ ^47^ and pyrethroids in 1990.^17^ The intensive and extensive use of these insecticides in public health and agriculture has exerted selective pressure on the mosquito populations, resulting in the development of resistance to various insecticides in *Ae. aegypti* and other mosquito vectors throughout the world.^48^ ^,^ ^49^ ^,^ ^50^ ^,^ ^51^ The present study reflects this phenomenon, in particular the population from Las Brisas, a neighborhood of Maracay city, where resistance to malathion, permethrin, and deltamethrin was detected (Table I). This city has consistently presented a high prevalence of dengue which has historically required intensive use of malathion and pyrethroids, although the vector control program has had very limited activities since 2010; the use of malathion was discontinued and instead only fenitrothion and temephos were used on a very restrictive basis. Resistance to malathion has been reported previously from the Maracay area^17^ ^,^ ^52^ ^,^ ^53^ as well as from other regions in Venezuela associated with metabolic resistance due to high levels of α- and ꞵ-esterases.^17^ ^,^ ^18^ ^,^ ^24^ ^,^ ^52^ Although we could not determine the levels of esterases in the present study, the moderate reversion of mortality with the use of the synergist PBO (Table I) suggests that enzymes such as oxidases (MFOs) might be partially involved in the metabolism of malathion. Elevated activity of GSTs was also detected in this population (Fig. 2). These mechanisms were also present, but to a lesser extent, in the population from Urbanización 19 de Abril which was susceptible to malathion (Table I). This suggests caution towards the future use of malathion in these localities, given the resistance detected in two of the sites and the detection of mechanisms associated with OP resistance in the third.

Although resistance is a focal phenomenon, it is widespread in Venezuela and has evolved rapidly.^18^ ^,^ ^19^ ^,^ ^21^ ^,^ ^52^ ^-^ ^54^ The first report of resistance to pyrethroids in Venezuela was to permethrin in mosquitoes collected in Maracay and Coro during 1992.^17^ A few years later, Pérez and Molina de Fernández^55^ detected resistance to lambdacyhalothrin in three populations from Aragua State, while a population from Girardot was also resistant to deltamethrin and a population from Mario Briceño Iragorry to cyfluthrin, with MFOs implicated as the primary mechanism of resistance. In the present study, resistance to permethrin and deltamethrin in populations of *Ae. aegypti* from Maracay was detected, even though these insecticides have not been used by the local control programs since 2010. Rodríguez et al.^54^ associated resistance to deltamethrin in *Ae. aegypti* from Venezuela and other Latin American countries with the activity of esterases, and GSTs, as well as cytochrome oxidases.^56^ We found significantly high levels of GSTs in all the populations, particularly the one from Las Brisas, which could be contributing to pyrethroid resistance (Fig. 2C). The levels of MFOs were significantly higher in the populations from Maracay (Las Brisas and Urbanización 19 de Abril) compared to the more recently established neighborhood (Nacupay) and susceptible (Rockefeller) strains, hence suggesting a role of MFOs in the pyrethroid resistance detected in the Maracay populations (Table I).

It is important to point out that resistance to pyrethroids might be due, to some degree, to cross-resistance with DDT.^57^ ^,^ ^58^ Both compounds share the same target site, the VGSC, and *kdr* mutations in this gene have been associated with changes in sensitivity in several insect groups, including *Anopheles* ^59^ ^,^ ^60^ and *Ae. aegypti* mosquitoes.^58^ ^,^ ^61^ ^,^ ^62^ ^,^ ^63^ The present study also detected point mutations in the gene coding for the VGSC (V1016I, F1534C, V410L), which have been previously reported in this species.^25^ ^,^ ^41^ ^,^ ^42^ ^,^ ^64^ The *kdr* mutations could be an additional mechanism contributing to pyrethroid resistance in the populations evaluated (Table III).

The *kdr* mutation V1016I was reported for the first time in four populations from Venezuela by Saavedra-Rodríguez et al.^25^ The V1016I mutation was also identified in mosquitoes collected in western and central Venezuela between 2008 and 2012^26^ and La Pedrera, Maracay, during 2012-2013,^21^ the latter located within 2 km from Las Brisas. In general, the frequencies reported for the mutant genotype were low (Table III), although higher than those reported previously by Saavedra-Rodríguez et al.^25^ Nevertheless, so far this mutation *per se* has not been confirmed to reduce VGSC sensitivity to pyrethroids.^41^ The *kdr* mutation F1534C has a wide geographic distribution in Asia, Africa, and Latin America.^14^ It has been demonstrated that this mutation reduces the VGSC sensitivity to pyrethroids^65^ and usually the mutant type has much higher frequencies than other co-occurrent mutations such as V1016I and V410L.^26^ ^,^ ^66^ ^,^ ^67^ ^,^ ^68^ In the present study, frequencies varied from 0.58 in Nacupay (population susceptible to Type I and Type II pyrethroids) to fixation with a frequency of 1.0 in Urbanización 19 de Abril (population resistant to permethrin but susceptible to cypermethrin) (Tables I, III, Fig. 1). The *kdr* mutation V410L was reported for the first time in *Ae. aegypti* from Brazil and confirmed reduced VGSC sensitivity to pyrethroids.^64^ The present study reports for the first time the presence of this mutation in *Ae. aegypti* populations from Venezuela. The frequencies observed were very low, particularly in the Nacupay pyrethroid-susceptible population (Tables I, III). Although at low frequencies, the strains from Las Brisas and Urbanización 19 de Abril had triple (LL/CC/II) and/or double (LL/CC or CC/II) homozygous mutants (Fig. 1). Although *kdr* alleles contribute to pyrethroid resistance, the elevated levels of metabolic enzymes might also play a key role.

It is important to point out that in the present study different generations were assayed. For the populations from Maracay (Las Brisas and Urbanización 19 de Abril), F1 and F2 were used, while for Nacupay we had F6 and F7. This fact might partly explain the susceptibility of *Ae. aegypti* in this area to all pyrethroids, as the loss of resistance can occur after several generations of colonization. However, this population notably remained resistant to malathion. It has been shown in laboratory studies that phenotypic resistance to pyrethroids can be lost after several generations free of insecticide pressure.^69^ ^,^ ^70^ ^,^ ^71^ Also, the Nacupay population showed the lower frequencies of the *kdr* alleles F1534C and I1016V. Nevertheless, results from the literature are inconsistent when analyzing the effect of colonization on the frequency of mutant *kdr* alleles. In fact, Vera-Maloof et al.^71^ showed that the frequencies of the alleles F1534C and I1016V declined after eight generations free from pyrethroid pressure although the rate and pattern of decline varied among strains. Grossman et al.^70^ showed significant loss of phenotypic resistance after ten generations while the double-mutant haplotype (CC/II) did not vary significantly over time.

The present study shows that despite the absence of strong insecticide pressure due to the very limited vector control operations since 2010, resistance persisted in *Ae. aegypti* populations. Similar results were found in Brazil^72^ where the use of pyrethroids for the control of *Ae. aegypti* was discontinued in 2000 due to high levels of resistance; those authors attributed the persistence of pyrethroid resistance, in part, to the personal use of insecticides by householders and private companies. This does not seem to be the case for Venezuela, as the availability of insecticides for personal use has been very limited and expensive due to the economic crisis and the private importation of insecticide was banned.

As stated earlier, insecticide resistance is a focal phenomenon: Molina de Fernandez et al.^20^ showed that within a few kms *Ae. aegypti* susceptibility to insecticides varied. Furthermore, spatial and seasonal fluctuations in insecticide resistance can be based on variation in the biology and genetics of vector populations.^73^ ^,^ ^74^ ^,^ ^75^ ^,^ ^76^ The results of the present study as well as those of Bastidas et al.^21^ indicate that resistance can be maintained for longer periods than expected in the absence of known insecticide pressure. One possible explanation is the limited gene flow among *Ae. aegypti* populations in Venezuela even though dispersion of *Ae. aegypti* eggs/adults can be easily carried out by different means of transportation.^77^

Insecticide resistance mutations have emerged independently all over the world, and the complexities of metabolic resistance implicate the overexpression of multiple genes.^78^ The increasing reports of insecticide resistance in *Ae. aegypti* populations all over the world is a clear signal to vector control programs and public health authorities about the importance of the rational use of insecticides coupled with alternative strategies. Also, there is an urgent need for investment in infrastructure for a reliable supply of piped water and solid waste disposal. A longitudinal study conducted in six municipalities of the Metropolitan Area of Maracay showed that all immature *Aedes* indices (house, container and Breteau) were very high and significantly related to the lack of reliable water supply,^79^ and irregular service of garbage collection and disposal was significantly related with an increase in dengue cases.^80^

As insecticide resistance continues to grow, the need for successful vector-borne disease control will require well-resourced, effective, and sustainable interventions and infrastructure that is less permissive of mosquito breeding.
